# Gene Editing and Genotoxicity: Targeting the Off-Targets

**DOI:** 10.3389/fgeed.2020.613252

**Published:** 2020-12-10

**Authors:** Georges Blattner, Alessia Cavazza, Adrian J. Thrasher, Giandomenico Turchiano

**Affiliations:** Infection, Immunity and Inflammation Research and Teaching Department, Zayed Centre for Research into Rare Disease in Children, Great Ormond Street Institute of Child Health, University College London, London, United Kingdom

**Keywords:** gene editing, CRISPR, genotoxicity, off-target, DSB = double-strand break, DNA damage, translocation, chromosomal aberration

## Abstract

Gene editing technologies show great promise for application to human disease as a result of rapid developments in targeting tools notably based on ZFN, TALEN, and CRISPR-Cas systems. Precise modification of a DNA sequence is now possible in mature human somatic cells including stem and progenitor cells with increasing degrees of efficiency. At the same time new technologies are required to evaluate their safety and genotoxicity before widespread clinical application can be confidently implemented. A number of methodologies have now been developed in an attempt to predict expected and unexpected modifications occurring during gene editing. This review surveys the techniques currently available as state of the art, highlighting benefits and limitations, and discusses approaches that may achieve sufficient accuracy and predictability for application in clinical settings.

## Introduction

Therapeutic approaches relying on the genetic engineering of cells for the treatment of hereditary diseases has long been a promising strategy to overcome the shortcomings of conventional drug therapies. The principle of these gene therapies is to counteract, correct, or replace a malfunctioning gene within cells that are most severely affected by the caused condition. However, any process affecting DNA integrity or causing DNA or chromosomal damage bears the risk of genotoxicity (Bohne and Cathomen, 2008).

While viral vectors utilized for gene addition-based strategies showed encouraging initial results (Anderson, 1990; Rosenberg et al., 1990; Gaspar et al., 2004), subsequent trials targeting hematopoietic stem and progenitor cells (HSPCs) exposed the risk of therapy-related toxicities, particularly insertional activation of proto-oncogenes leading to malignant cell transformation. Indeed, the activation of MDS1-EVI1 and LMO2 oncogenes caused by the integration of the gamma retroviral vector led to clonal skewing and development of malignancies in patients enrolled in several gene therapy clinical trials. (Hacein-Bey-Abina et al., 2003; Raper et al., 2003; Ott et al., 2006; Cattoglio et al., 2007; Schwarzwaelder et al., 2007; Howe et al., 2008; Metais and Dunbar, 2008; Stein et al., 2010; Zhou et al., 2016). These issues have been partly addressed through development of next generation vectors including in particular lentiviruses (Naldini et al., 1996; Aiuti et al., 2013; Hacein-Bey-Abina et al., 2014; Kohn et al., 2020) and adeno-associated virus (AAV) (Nathwani et al., 2011) lowering, but not eliminating, the risk of insertional mutagenesis and immunogenicity.

Besides the risk of insertional mutagenesis and immunogenicity (Cavazzana-Calvo et al., 2010), viral vectors have additional drawbacks, including their inability to address dominant mutations and their potential influence on the host cell's gene expression (Maeder and Gersbach, 2016). Further attempts to address these issues have been made, for example by using chimeric proteins to retarget lentiviral integration to sites with reduced transcriptional activity (Gijsbers et al., 2010; Vranckx et al., 2016).

Many of these limitations can however be overcome by gene therapy approaches that rely on genome editing techniques which enable more precise, targeted genomic modifications to restore wild-type sequences, while preserving the temporal and tissue-specific control of the afflicted gene, or to specifically knock out genes.

Initially the four main families of nucleases–meganucleases (Chevalier et al., 2001; Epinat et al., 2003), zinc finger nucleases (ZFNs) (Urnov et al., 2005), transcription activator-like effector nucleases (TALENs) (Bogdanove and Voytas, 2011), and Clustered Regularly Interspaced Short Palindromic Repeats (CRISPR)-associated nucleases (Cas) (Jinek et al., 2012) were used to induce targeted DNA double-strand breaks (DSBs). Meganucleases, ZFNs, and TALENS are tethered toward specific DNA sequences by means of DNA-binding protein domains while the CRISPR-Cas system is based on a nuclease protein guided by an RNA molecule complementary to the targeted DNA sequence (gRNA) via Watson-Crick base pairing. The introduction of DSBs activates one of the two main endogenous cellular repair pathways, including the error prone non-homologous end joining (NHEJ) and the homology directed repair (HDR) pathways. Some repurposed derivatives of the engineered nucleases, in particular of Cas, have been developed to fulfill different tasks. Nickases, which are Cas9 proteins with only one functional nuclease domain (Sapranauskas et al., 2011; Jinek et al., 2012), are used to induce DNA single-stranded breaks (SSBs), combined with base editors (BEs) fused to a cytidine or an adenine deaminase to induce precise transition mutations (Komor et al., 2016, 2017; Gaudelli et al., 2017; Kurt et al., 2020). In addition, the prime editing strategy uses a nickase fused with a reverse transcriptase complexed with a prime editing guide RNA (pegRNA) to mediate targeted insertions of few bases, deletions, and base conversions (Anzalone et al., 2019).

Similar to viral vectors, genome editing techniques have been rapidly adopted and they have proven suitable for clinical application in various fields. So far, seven patients have been infused with CRISPR-Cas9 modified autologous CD34+ HSPCs for the treatment of beta-hemoglobinopaties, showing encouraging results; among those, two patients affected by beta thalassemia are transfusion independent after 5 and 15 months after infusion while one patient affected by sickle cell disease is free of vaso-occlusive crises at 9 months after treatment (NCT03655678; NCT03745287). CRISPR-Cas9 or TALENs have also been applied to engineer patient or Universal Chimeric Antigen Receptor (CAR)T lymphocytes for improved antitumor immunity (Qasim et al., 2017; Stadtmauer et al., 2020) (NCT02735083; NCT02808442; NCT02746952; NCT03081715; NCT02793856; NCT04244656; NCT04035434). Moreover, TALENs have been used to create allogenic CS1, CD123, or CD22-specific CAR-T cells (NCT04142619; NCT03190278; NCT04150497) and ZFNs helped to engineer T cells with a C-C motif chemokine receptor 5 knockout to induce resistance to HIV infection (Tebas et al., 2014) (NCT03617198). In addition, AAV vectors together with ZFN-mediated genome editing were applied for the insertion of a correct copy of the α-L-iduronidase gene for subjects with attenuated Mucopolysaccharidosis type I (MPS I) (NCT02702115).

While genome editing techniques address certain limitations and reduce particular risks of genotoxicity in viral vector-based gene therapy, they entail new complications. Off-target activity, the induction of DNA modifications at unintended sites, is a concern with all designer nucleases (Fu et al., 2013; Koo et al., 2015). Such off-target activity can potentially lead to point mutations, deletions, insertions or inversions. Besides off-targeting due to a sequence similarity to the targeted site, there can also be collateral cleavage activity. This has been observed for CRISPR-Cas12a, which, upon RNA-guided on-target DNA binding, non-specifically cleaves single-stranded DNA molecules (Chen et al., 2018). While high fidelity variants of the Cas9 protein have successfully been developed to reduce off-target activity (Kleinstiver et al., 2016; Vakulskas et al., 2018) they still bear the risk of inducing on-target damage after cleavage in the form of large deletions spanning several kilobases or translocations (Kosicki et al., 2018; Connelly and Pruett-Miller, 2019; Turchiano et al., 2020). As those large deletions can bring relatively distant elements close together, they could have a genotoxic potential similar to the insertional mutagenesis caused by viral vectors. A key prerequisite for the clinical application of genome editing tools is the monitoring of their safety before, during and after the administration of the treatment. However, while gene therapy and genome editing are advancing at a rapid pace, the application of appropriate assays to evaluate unintended genomic effects suffers from a lack of standardized methods and guidelines (Corrigan-Curay et al., 2015). A multitude of techniques have been developed in the recent years to detect small insertions and deletions (Indels), potential off-target DNA breaks, translocations, or viral integration sites but the lack of standardized analyses that allow an absolute quantification of those modifications makes a direct comparison among these tools cumbersome. The aim of this review is to give an overview of the drawbacks and benefits of the currently available tools to assess the safety of gene editing applications and of the parameters that need to be taken into account for a correct safety assessment of a gene therapy approach.

## Biased Detection Methods

A major step for the successful use of designer nucleases is the choice of the target site and the according nuclease or gRNA design following criteria of editing efficiency and specificity. Potential off-targets can be predicted *in-silico* (Grau et al., 2013; Bae et al., 2014; Cradick et al., 2014; Montague et al., 2014; Concordet and Haeussler, 2018) or identified on cultured cells (in cellula) or *in vitro* assays for DSB detection. The list of potential off-target sites identified must be subsequently verified in the target cell type/tissue alongside the detection of on-target cleavage. There are three common types of approach to quantify indels formation at the selected sites, all of which rely on polymerase chain reactions (PCR) performed on genomic DNA treated with the designer nuclease of choice. After denaturation and rehybridization of the PCR products, hetero-duplex DNA containing a mutated and a wild-type strand can form, which can then be cleaved by mismatch-detection nucleases such as Surveyor nuclease or T7 endonuclease I (Mashal et al., 1995; Qiu et al., 2004), enabling the quantification of the cleavage products by electrophoresis. While cost-effective and simple, a drawback of those techniques is that single nucleotide polymorphisms are poorly recognized. Quantification of Indels in the PCR products can also be determined by methods such as Tracking of Indels by Decomposition (TIDE) or Inference of CRISPR Edits (ICE) analyses, which compare the Sanger sequence chromatograms of an untreated control against a treated sample at the intended editing site (Brinkman et al., 2014; Hsiau et al., 2019). Moreover, Indels can also be directly quantified using deep sequencing of the PCR products (Pinello et al., 2016). As all of these approaches are based on PCR amplicons of around 200–700 base pairs (bp) from the potential target loci, they all suffer from the same shortcoming, that is the missed detection of larger deletions or other aberrations that could encompass at least one of the PCR primer binding sites. While it is commonly accepted that most of the indels fall in a size spectrum of under 50 bp (Koike-Yusa et al., 2014; van Overbeek et al., 2016), it has been shown that genome editing can also lead to large deletions of several kilobases (kb) (Kosicki et al., 2018; Chakrabarti et al., 2019; Turchiano et al., 2020). Moreover, even in the case of deletions being amplified/detected by PCR, a minimal sequence length of sufficient quality, required for the alignment with either a control sequence or a reference genome, might not be reached.

Indel assessment by deep sequencing requires additional consideration. Artifactual sequencing errors produce a background signal that is usually filtered out by setting an arbitrary threshold to define the relevant modified loci. This kind of analysis can produce either false-positives or false-negatives since every amplicon can present higher or lower background levels, respectively. Statistically more robust approaches are needed, but they can be laborious particularly when a multitude of targets are investigated. Performing the analysis on a large set of replicates and untreated controls in order to compare the mean editing frequencies using a *t*-test would be the preferable procedure (Zeng et al., 2020). Alternatively a two-sample test for equality of proportions or a Fisher's exact test can be performed to detect differences between the mutation rates of edited and untreated samples. In order for this approach to be robust, a high number of reads is desirable. Moreover, when different assays are employed, these statistical tests can account for the variability of the NGS measurements to better define the null hypothesis or introduce a false discovery rate correction (e.g., Benjamini–Hochberg; Kuscu et al., 2014; Turchiano et al., 2020). This would be a more traceable practice compared to indel values subtractions between the treated and the relative untreated samples (Cameron et al., 2017) or to simply assuming a background noise level (Yang et al., 2014; Kim et al., 2016; Kim and Kim, 2018). This approach allows compensation for sequencing errors, especially for challenging regions with repetitive elements, which might produce a considerable amount of indel-like reads and that might even require a visual inspection to evaluate potential sequencing or alignment artifacts (Zeng et al., 2020).

Alternatively, oligo integration analysis rather than indel detection allows dramatic reduction in background noise in the untreated control of about 100 fold, enabling off-target detection at <0.001% rates (Tsai et al., 2017). It is worth noting that the reliability of this method is dependent on the oligo integration efficiency in the cell type of interest, generally higher in cell lines as compared to primary cells; moreover this technique cannot be applied to samples that are meant to be infused into patients, since this would imply integrations of non-therapeutically relevant exogenous sequences into the genome.

## Unbiased Detection Methods

### DSB Detection

While *in silico* off-target prediction is a fast and cheap option, it suffers from high false-positive rates as it is mostly based on the similarity of a sequence to the target site and does not consider differences due to genetic variants; moreover, it has a limited sensitivity in the detection of bona fide off-targets (Tsai et al., 2015; Kim et al., 2019b). This bias can be overcome with the use of *in vitro* methods that are based upon the incubation of purified genomic DNA with the designer nuclease of choice. The DSBs induced by the nuclease are then detected in various ways, either by the circularization of the created DNA fragments in CIRCLE-seq (circularization for *in vitro* reporting of cleavage effects by sequencing) or CHANGE-seq (circularization for high-throughput analysis of nuclease genome-wide effects by sequencing) (Tsai et al., 2017; Lazzarotto et al., 2020), the ligation of adapters in SITE-seq (selective enrichment and identification of adapter-tagged DNA ends by sequencing) (Cameron et al., 2017) or End-seq (DNA end sequencing) (Canela et al., 2016), or deep sequencing and identification of identical 5' DNA fragments in Digenome-seq (*in vitro* Cas9-digested whole-genome sequencing) (Kim et al., 2015). While being sensitive, a common drawback of these approaches is a tendency to overestimate the number of sites that are actually modified in cells (Cho et al., 2014), as the influence of the chromatin structure in determining the DNA accessibility is widely disregarded (Kim and Kim, 2018). Moreover, the impact of the nuclease concentration inside the cell (Wu et al., 2014) and of the delivery method on the cleavage footprint are not considered by *in vitro* assays (Kim et al., 2014; Cameron et al., 2017). Those *in vitro* techniques are usually returning the highest number of sites, but their relative validation rates disregard at least half of them in the best case scenario. The *in cellula* derived deep sequencing validation deserves some additional considerations: (1) it is usually performed only on the top performing sites disregarding the ones close to the cutoff thresholds; (2) it cannot be sensitive enough to detect rare indel events; (3) some DSBs can be perfectly repaired without creating any mutation and therefore could be missed during the validation process.

A more representative assessment can hence be expected from methods where designer nucleases are applied directly in cellula. In GUIDE-seq (genome-wide, unbiased identification of DSBs Enabled by sequencing; Tsai et al., 2015), IDLV (integrative-deficient lentiviral vectors) capture (Gabriel et al., 2011) and ITR-seq (Inverted Terminal Repeat sequencing; Breton et al., 2020) the DSBs are marked by insertion of exogenous sequences, which are subsequently exploited as specific primer binding sites and then amplified via linker mediated PCR. Instead techniques like BLESS (direct *in situ* breaks labeling, enrichment on streptavidin and next-generation sequencing; Crosetto et al., 2013; Ran et al., 2015), its variant DSB Capture (Lensing et al., 2016) or BLISS (Breaks Labeling *in situ* and Sequencing; Yan et al., 2017, 2019) are based on *in situ* processing of the DNA at the open DSB ends and ligation of biotinylated adapters or adaptors for *in vitro* transcription. For DSB-seq high molecular weight genomic DNA is isolated from treated cells and the DNA ends are 3'-end tailed with biotinylated nucleotides by terminal deoxynucleotidyl transferase (TdT) before sonication, capturing, and sequencing (Baranello et al., 2014). An alternative approach based on Chromatin immunoprecipitation sequencing (ChIP-seq) targets the phosphorylated histone variant H2A.X or other repair factors that are recruited to cleaved sites (Iacovoni et al., 2010). However, as those factors can spread several kb around DSBs, an identification of the cleavage sites at nucleotide resolution is difficult. In DISCOVER-seq (discovery of *in situ* Cas off-targets and verification by sequencing), detection of the MRE11 subunit of the MRN complex binding by ChIP-seq returns a more specific and sensitive information filtered by a custom algorithm that retains cleaved sites followed by the protospacer-adjacent motif (PAM) and the putative protospacer binding site (Wienert et al., 2019, 2020). It is worth mentioning here that unbiased DSB discovery is also performed by some of the techniques described in section “Translocation and Other Chromosomal Aberration Detection.”

### SSB/BE Detection

Compared to the variety of assays for DSB detection, methods to monitor SSBs induced by designer nickases and/or base editors are less abundant. This type of gene editors is generally thought to be less harmful than designer nucleases generating DSBs (Hu et al., 2016; Bothmer et al., 2017) but thorough and more specific analyses could report a higher genome and RNA mutational rate with some BEs (Rees et al., 2019; Xin et al., 2019).

Detection techniques designed for and tested on samples treated with designer nickases linked to BEs are EndoV-seq (Endonuclease V-based sequencing; Liang et al., 2019) and Digenome-seq (Kim et al., 2017, 2019a, 2020) (Supplementary Table 2). Both techniques rely on an *in vitro* nicking, base modification and subsequent DNA end repairing in order to obtain a particular pattern after whole genome sequencing (WGS). This approach and its respective bioinformatic analysis help to filter out the majority of the natural occurring DNA nicks, but requires a good WGS coverage (>30 ×), which makes these techniques expensive and only applicable to studies in a pre-clinical phase.

The validation rate for these techniques can vary greatly compared to DSB detection methods. Since SSBs cannot be directly revealed by indel quantification, base editing frequencies at potential off-target sites can be measured by NGS or, in alternative, DSBs induced by an active designer nuclease can be employed as surrogates.

Other techniques are showing great potential but they have not been tested on designer editors/nickases. Among those, SSB-seq (Baranello et al., 2014), SSiNGLe (single-strand break mapping at nucleotide genome level) (Cao et al., 2019), GLOE-seq (genome-wide ligation of 30-OH ends followed by sequencing) (Sriramachandran et al., 2020), and Nick-seq (Cao et al., 2020) are able to return data from an in cellula approach.

The “Prime editors” strategy instead presents a new challenge for this kind of techniques since its nickase activity is coupled with a reverse transcriptase that could potentially introduce indels at off-target sites or cause retrotransposon activations and integrations of random reverse transcribed RNA sequences into the genomic DNA (Anzalone et al., 2019). A recent work using Digenome-seq (Kim et al., 2020) exploited the aspecific capacity of the dCas9-H840A protein, utilized in the PE, to cleave also the non-targeted strand, resulting in a characteristic signature after WGS and enabling the use of an analysis compatible with the Digenome-seq bioinformatic pipeline. The authors showed that not all the off-target sites detected by Digenome-seq and validated for the presence of indels are prime-edited, confirming the importance of the pegRNA specific priming activity. In support of this, a different work recently showed the presence of unexpected large deletions after prime editing in mice embryos, mainly ascribed to the dCas9-H840A activity (Aida et al., 2020). However, this proposed strategy do not allow the detection of all the possible mutations that this system may induce *in cellula* and therefore it is not completely exhaustive.

### Translocation and Other Chromosomal Aberration Detection

Off-target mutations and insertional mutagenesis are considered to be harmful because they can perturb the expression of nearby genetic elements by means of different mechanisms (McCormack and Rabbitts, 2004). While this dysregulation is usually localized, rare or innocuous, major concerns derive from general genomic instability and the several chromosomal aberrations that we may or may not detect after editing. Increasing evidences are showing how those gross chromosomal aberrations generate after designer nucleases activity (Weinstock et al., 2008; Kosicki et al., 2018; Turchiano et al., 2020). Oncongenic translocations have been reproduced *in vivo* in lung tissues of mouse models by the simultaneous introduction of two DSBs, confirming this major concern (Blasco et al., 2014). Large deletions, loss of heterozygosity, large inversions, or translocations may also impact the 3D genomic organization and cause dysregulations of entire topological associated domains (median size ~880 kb) usually organized to be transcriptionally active or repressed (Dixon et al., 2012; Bonev and Cavalli, 2016).

Recent studies also observed on-target related chromosomal aberrations with formation of micronuclei and chromosome bridges leading to copy number variation, telomeric portion loss, and chromotripsis (Cullot et al., 2019; Leibowitz et al., 2020) rising further concerns for the safety of designer nucleases in clinic.

The portfolio of translocation detection techniques developed to recognize those mutations with increasing sensitivity comprises TC-seq (translocation capture sequencing; Klein et al., 2011), UDiTaS (UniDirectional Targeted Sequencing; Giannoukos et al., 2018), AMP-seq (anchored multiplexed PCR sequencing; Zheng et al., 2014), LAM-HTGTS (linear amplification-mediated high-throughput genome-wide sequencing; Frock et al., 2015), and CAST-seq (chromosomal aberration analysis by single targeted LM-PCR sequencing; Turchiano et al., 2020).

All of these methods are based on nested PCRs with primers binding between a known target site and fused unknown sites marked by an adapter. NGS sequencing is then used to identify the fusion partners. Differences among these techniques include in particular the adapter attachment via tagmentation (UDiTas), bridge adaptor ligation (LAM-HTGTS), or dsDNA ligation (CAST-seq and AMP-seq). Besides, the amount of input DNA and the bioinformatic pipeline can differ substantially for these techniques as shown in Supplementary Table 3. For designer nucleases, the validation of translocation sites can be performed by looking for off-target cleavage at the fused sites by deep sequencing. On the other hand, translocations can also be directly validated through PCR or ddPCR with specific primers recognizing the two fusion partners (Bak et al., 2017). As translocations themselves exhibit a distinguishable element in the form of the specific point of fusion, the quantification of individual events becomes easier even without the addition of unique molecular identifiers (UMIs) barcodes.

## Technical Challenges

All of these techniques have potential drawbacks in their methodology or bioinformatic analysis as summarized in Supplementary Tables 1–3 (excluding techniques not optimized to describe designer nucleases activity). Besides the above mentioned biological biases, the distinction between *in vitro* and *in cellula* assays is essential due to their expected difference in sensitivity and hence in the number of returned off-target sites; for *in vitro* techniques, the introduction of an arbitrary threshold (e.g., the amount of reads per element) to select a smaller subset of sites could be an option, even though it may introduce a significant bias in the off-target detection. In a clinical setting, this bias could be reduced by further validating some of the sites that have been discarded by the arbitrary threshold, as for example those that are found close to oncogenic elements within a window of 50–100 kb of distance.

Another aspect that is crucial from the perspective of clinical application is the required amount of DNA or cell input, since attainable patient samples are limited. Some techniques might not be suitable for the analysis of the most clinically relevant samples. The introduction of additional elements (IDLV, DNA oligonucleotides) into the cell makes approaches like GUIDE-seq and IDLV capture unsuitable for performing the off-target screening directly on the cells intended for the treatment. When using these techniques, off-target detection must be performed in surrogate cell lines hence the cleavage footprint might not be accurate for a particular patient or the particular treatment due to diverging sequences, chromatin state, or DNA accessibility.

While the qualitative description of the off-target sites is an important information, their cleavage frequencies, and their reliable ranking can be equally of value, especially when monitoring the clonal expansion of modified cells in patients. Barcode sequences can be introduced at the cleavage site or upon adapter ligation prior to amplification steps in order to quantify individual events in an unambiguous manner. On the other hand, a semiquantitative/quantitative information might still be retrieved, without barcoding, by calculating the relative reads amount of a certain mutation over the total amount of reads (Crosetto et al., 2013; Wienert et al., 2019) or by utilizing other unique molecular signatures such as the linker ligation point and the translocation fusion point (Zheng et al., 2014; Frock et al., 2015; Turchiano et al., 2020).

Not least the bioinformatic pipelines are of major importance due to the potential biases they can introduce or remove. Sequences filtering process is mainly borne by the reads/alignments quality and the reads amount counted in a defined genomic region. In order to avoid false positive results, the comparison with an untreated control can be beneficial, as it limits the biases coming from sequence misalignment or indexing hopping phenomena (Kircher et al., 2012) in multiplex NGS. A problematic practice is filtering out sites that do not reach a defined degree of homology to the on-target site. This kind of filtering, sometimes arbitrarily defined, can have a particular impact in case of differences between the patient's and the reference genome. Additionally, unspecific cleavage phenomena such as collateral activity of Cas12a (Li et al., 2018) would never be observed with this kind of filtering. This approach can be difficult to be applied when using other heterodimeric designer nucleases such as TALENs or ZFNs, especially if homodimers or other unintended dimers orientations and distances that can lead to cleavage are considered.

The parameters that may be used to define the potency of an assay could be the sensitivity and the accuracy. For a well-founded sensitivity assessment a known amount of potentially detectable events is ideally present within a sample or where a specific detected event can be quantified by other means and tested in a dilution series. Estimating the sensitivity based on measurements of indels for example suffers from the aforementioned uncertainty of the NGS analysis itself. It is also worth noting that the sensitivity of a technique depends also on the experimental conditions, and could be directly proportional to the numbers of cells treated, the amount of input material and the sequencing depth. The accuracy of this kind of technique relies on the ability to detect off-targeted sites with a minimal error rate or bias and is dependent by the amount of validated false positive and false negative sites. The validation process usually relies on deep sequencing of the inquired genomic regions to discern the false positive sites with all the drawbacks beforehand described. Supplementary Tables 1–3 reports the validation rates for the described techniques; however we do not have an objective and complete overview of all the mutations induced by the genome editing tools therefore calculating the false negative rate parameter is impossible with the current state of the art.

In this scenario, we also have to include the possibility of a designer nuclease having off-target activity in some genome widespread repetitive elements. Genomic instability events in these regions would be worrisome as they can be abundant, difficult to align and might be filtered out by the relative bioinformatic pipelines. In this case, use of unmasked reference genomes and a tolerant alignment algorithm together with the comparison with the untreated control would help mitigating the bias and finding a balance with the accuracy rate.

## Conclusions and Outlooks

Gene therapy based on integrating viral vector evolved in the last decades together with techniques and analyses that can at least in part evaluate safety (Modlich et al., 2006; Montini et al., 2006; Zhou et al., 2016; Biasco, 2017). In the same way, the different gene editing strategies are shaping novel reagents, techniques and strategies to improve their safety and efficiency for clinical application (Miller et al., 2007; Kleinstiver et al., 2016; Casini et al., 2018; Gao et al., 2018; Vakulskas et al., 2018; Rai et al., 2020). Further studies are required to understand and analyse the genotoxicity of new therapeutic strategies, and compare it with existing technologies. So far, the genotoxic effects of retroviral vectors employed in gene therapy approaches have been linked to insertional mutagenesis events mediated by viral enhancers (Bohne and Cathomen, 2008; Hacein-Bey-Abina et al., 2014), while designer nucleases act differently and may be more detrimental in regards to cell viability and genome integrity (Schiroli et al., 2019; Leibowitz et al., 2020). Delivery methods were also shown to impact differently on the mutational capacity of designer nucleases and the scientific community is moving toward a hit-and-run approach, utilizing ribonucleoproteins, or mRNA, that ensures a quick clearance of the exogenous nuclease and a more specific activity (Hendel et al., 2015). A balanced discussion should also consider the different impact of mutations in stem or in differentiated cells, with the latter likely to bear a lower risk of genotoxicity due to their shorter lifespan.

In light of these observations we can derive a new definition for genotoxicity which can be described as the property of an agent able to alter the genetic function within a cell causing unwanted mutations/effects, which may lead to functional impairment (e.g., cancer, therapy impairment, differentiation impairment).

CRISPR-Cas technology has largely democratized and accelerated the gene editing field but there are not yet standard techniques that can evaluate all the possible mutations induced directly or indirectly by the editing procedure. Recent publications revealed that off-targeting is responsible only for a minor portion of mutations characterized in edited cells, while there are on-target related mutations that justify careful evaluation (Kosicki et al., 2018; Connelly and Pruett-Miller, 2019; Cullot et al., 2019; Turchiano et al., 2020).

Hence, a combination of an *in cellula* technique for the discovery of off-targets sites (DISCOVER-seq, BLISS) and one for all other chromosomal aberrations (CAST-seq, HTGTS) would likely detect most of the unexpected mutations without the need to modify the current ex-vivo clinical procedures. *In vitro* techniques can describe a worst-case-scenario of off-target editing in a pre-clinical setting but require in addition a thorough validation via deep sequencing to exclude the abundant false positive sites that can be returned by the analysis, even when using base editors (see Supplementary Tables 2, 3). In clinical settings, where hundreds of millions of cells need to be edited, the deep sequencing indel detection threshold of 0.01–0.1% may not be sufficient to detect the actual off-target activity. CAST-seq shows an increased sensitivity reaching 0.006% (1 mutation out of 15,000 genome haplotypes) when compared with the absolute ddPCR quantification capacity, and hopefully also DSB detection techniques may be improved in the near future to achieve or lower that threshold in therapeutic settings.

In this review, we have highlighted the currently available techniques to detect DSBs, SSBs, translocations, and other chromosomal aberrations and the methods to quantify cleavage of designer nucleases. Overall, the amount of input DNA, the reliable quantification of events, an unbiased bioinformatic pipeline, the traceable sensitivity and the validation rate assessment are critical to evaluate the suitability of a technique.

The gene therapy field is moving fast; new molecular strategies are being proposed or are now under investigation in order to expand the applications and improve the editing efficiency. Prime editing (Anzalone et al., 2019), for example, could entail new potential. Alternatively, a new site specific and scareless integrative strategy could be developed soon by harnessing the transposase activity (Voigt et al., 2012; Klompe et al., 2019; Kovac et al., 2020), making another giant leap forward in the field. Advanced methods to assess genotoxicity of such technologies must be devised and will hopefully incorporate additional sensitivity and capacity to quantify all genetic modifications introduced by current and next-generation gene editing platforms.

## Author Contributions

GB, AC, AT, and GT contributed to the final version of the manuscript. All authors contributed to the article and approved the submitted version.

## Conflict of Interest

GT and GB have filed a patent application on CAST-seq. AT is on the Scientific Advisory Board of Orchard Therapeutics and Rocket Pharmaceuticals. The remaining author declares that the research was conducted in the absence of any commercial or financial relationships that could be construed as a potential conflict of interest.
